# Towards highly accelerated Cartesian time-resolved 3D flow cardiovascular magnetic resonance in the clinical setting

**DOI:** 10.1186/1532-429X-16-42

**Published:** 2014-06-18

**Authors:** Daniel Giese, James Wong, Gerald F Greil, Martin Buehrer, Tobias Schaeffter, Sebastian Kozerke

**Affiliations:** 1 Division of Imaging Sciences and Biomedical Engineering, King’s College London, London, UK; 2 Department of Radiology, University of Cologne, Cologne, Germany; 3 Institute for Biomedical Engineering, University and ETH Zurich, Zurich, Switzerland

**Keywords:** Cardiovascular magnetic resonance, Flow quantification, Phase-contrast CMR, 4D flow CMR

## Abstract

**Background:**

The clinical applicability of time-resolved 3D flow cardiovascular magnetic resonance (CMR) remains compromised by the long scan times associated with phase-contrast imaging. The present work demonstrates the applicability of 8-fold acceleration of Cartesian time-resolved 3D flow CMR in 10 volunteers and in 9 patients with different congenital heart diseases (CHD). It is demonstrated that accelerated 3D flow CMR data acquisition and image reconstruction using *k-t* PCA (principal component analysis) can be implemented into clinical workflow and results are sufficiently accurate relative to conventional 2D flow CMR to permit for comprehensive flow quantification in CHD patients.

**Methods:**

The fidelity of *k-t* PCA was first investigated on retrospectively undersampled data for different acceleration factors and compared to *k-t* SENSE and fully sampled reference data. Subsequently, *k-t* PCA with 8-fold nominal undersampling was applied on 10 healthy volunteers and 9 CHD patients on a clinical 1.5 T MR scanner. Quantitative flow validation was performed in vessels of interest on the 3D flow datasets and compared to 2D through-plane flow acquisitions. Particle trace analysis was used to qualitatively visualise flow patterns in patients.

**Results:**

Accelerated time-resolved 3D flow data were successfully acquired in all subjects with 8-fold nominal scan acceleration. Nominal scan times excluding navigator efficiency were on the order of 6 min and 7 min in patients and volunteers. Mean differences in stroke volume in selected vessels of interest were 2.5 ± 8.4 ml and 1.63 ± 4.8 ml in volunteers and patients, respectively. Qualitative flow pattern analysis in the time-resolved 3D dataset revealed valuable insights into hemodynamics including circular and helical patterns as well as flow distributions and origin in the *Fontan* circulation.

**Conclusion:**

Highly accelerated time-resolved 3D flow using *k-t* PCA is readily applicable in clinical routine protocols of CHD patients. Nominal scan times of 6 min are well tolerated and allow for quantitative and qualitative flow assessment in all great vessels.

## Background

Acquiring time-resolved whole heart 3D phase contrast cardiovascular magnetic resonance (CMR) with flow encoding in three spatial dimensions is limited by its intrinsically long scan times [1]. This results in trade-offs between spatial and temporal resolutions and/or tolerating potential breathing motion artefact when acquiring the data in a clinical routine setting [2]. However, the ability of time-resolved 3D CMR to assess flow volumes, pulse wave velocities [3], pressure gradients [4], wall shear stress [5-7] and turbulent kinetic energy [8,9] requires high spatial and temporal resolution. Knowledge about the high degree of redundancy in phase contrast velocity data has fuelled efforts to acquire Cartesian undersampled time-resolved data using parallel imaging or a combination of parallel imaging and spatiotemporal constraints [10-12]. These techniques have been limited by temporal blurring artefacts when using undersampling factors larger than 5 in both time-resolved 3D [13-15] and 2D phase-contrast CMR [11,16]. Recent improvements of reconstruction algorithms dedicated to phase-contrast imaging have enabled acceleration factors greater than 5 for 3D phase-contrast CMR of the carotid bifurcation [17]. Non-Cartesian time-resolved 3D flow CMR and non-linear constrained reconstruction techniques have also been proposed with net acceleration factors of 2-5 [18,19]. Especially due to the long scan times of 3D flow CMR, single slice through-plane phase-contrast imaging (time-resolved 2D flow CMR) in combination with parallel imaging often remains the method of choice for flow measurements in clinical protocols [20]. A 2D scan can be targeted to specific vessels and can be acquired during a breath-hold or during free shallow breathing with several signal averages to reduce breathing motion artefacts. Apart from only acquiring through-plane blood velocities in a single slice, a practical limitation of this technique relates to time-consuming slice planning, in particular in congenital heart disease (CHD) patients. Although the image analysis of 3D flow CMR remains time-consuming [21], the possibility of retrospectively adapting planes within the 3D volume is of great advantage [22].

In this work, highly undersampled Cartesian time-resolved 3D images were acquired covering the entire heart and surrounding vessels and reconstructed using the previously presented *k-t* PCA [23] algorithm in combination with a sparsifying transform [17]. The technique was applied on retrospectively undersampled data using different acceleration factors in order to allow direct evaluation of acceleration effects with respect to flow values derived from fully sampled data. In addition to determining optimal scan parameters for the prospectively undersampled acquisitions, the retrospectively undersampled data were compared to *k-t* SENSE reconstructions. Using a nominal acceleration factor of 8, the technique was then combined with respiratory gating and applied to 10 healthy volunteers and 9 patients with congenital heart diseases (CHD) on a clinical 1.5 T CMR scanner. Quantitative flow analysis was performed in vessels of interest and compared to time-resolved 2D through-plane flow acquisitions for validation. Particle trace visualisation was used to qualitatively assess flow patterns in patients.

## Methods

### Data acquisition

The study protocol was reviewed and approved by the institutional ethics committee (10/H0802/65)’ and written informed consent was obtained from all participants or their parents. Data were obtained on a 1.5 T Achieva System (Philips Healthcare, Best, The Netherlands) using a 5 channel array cardiac coil in volunteers and a 2-5 channel array coil in CHD patients (depending on patient size).

Similar to previous *k-t* acceleration validation studies [11,12], a reference 3D flow dataset with a *k-t* factor of 1 was first acquired (using standard SENSE x 2), resulting in a nominal scan time of 25 minutes. Scan parameters are listed in Table 1 and further included a flip angle of 6°, a repetition and echo time of 4.5 ms and 2.5 ms respectively and a velocity encoding range (v_enc_) of 250 cm/s. A symmetric four-point encoding scheme [24] was used for all time-resolved 3D flow acquisitions. This fully sampled dataset was used to investigate the accuracy of the presented *k-t* PCA reconstruction as compared to a *k-t* SENSE reconstruction. Different nominal *k-t* undersampling factors ranging from 2 to 12 were simulated.

**Table 1:** Vessels of interest and phase contrast acquisition parameters

**#**	**CHD**	**2D flow**	**3D flow**					**Age**	**GA**
		**Vessels of interest (v**_**enc**_**[cm/s])**	**Spatial res. [mm**^**3**^**]**	**# Phases (**Δ**t [ms])**	**FoV [mm**^**3**^**]**	**v**_**enc**_**[cm/s]**	**Scan time [min]**		
0	Volunteers	AAo (200), MPA (200), RPA (200), LPA (200), SVC (100)	2.5×2.5×2.5	24	320×320×140	200	5.6	29 y	no
1	HLHS (I)	AAo (200), LPA (150), RPA (200)	2.5×2.5×1.75	32 (17)	140×70×200	400	3.6	11 m	yes
2	ToF	AAo (200), MPA (150)	1.78×1.61×2.5	24 (33)	200×280×100	200	5.2	21 y	no
3	HLHS (II)	AAo (250), DAo (150), LPA (70), RPA (70), SVC (70)	1.26×1.61×1.79	24 (21)	141×180×79	200	5.2	2 y	yes
4	HLHS (III)	AAo (200), LPA (80), RPA (80), SVC (80), IVC (80)	2×1.4×2	24 (37)	208×320×130	150	7.1	11 y	yes
5	DILV (III)	AAo (200), LPA (80), RPA (80), SVC (80), IVC (80), Fen (150)	1.18×1.71×1.72	24 (24)	151×220×95	150	6.8	3 y	yes
6	HLHS (II)	AAo (200), Dao (200), LPA (80), RPA (80), SVC (80)	2.08×2.08×2.08	24	167×300×94	200	5.1	7 m	yes
7	HLHS (III)	AAo (150), Dao (150), LPA (80), RPA (80), SVC (80), IVC (80)	2.31×2.31×2.31	32	203×370×115	150	7.6	9 y	yes
8	HLHS (II)	AAo (200), Dao (200), LPA (80), RPA (80), SVC (80)	2.08×2.08×2.08	24	167×300×94	120	5.8	2.5 y	yes
9	HLHS (II)	AAo (200), Dao (200), LPA (80), RPA (80), SVC (80)	2.08×2.08×2.08	24	167×300×94	130	5.2	3 y	yes

Scan parameters. HLHS (I-III): Hypoplastic Left Heart Syndrome after step I, II or III of the Fontan procedure, ToF: Tetralogy of Fallot, DILV: Double Inlet Left Ventricle. Nominal scan times excluding navigator efficiency are given. GA: general anaesthesia.

In 10 healthy volunteers (mean age: 28.6 years, range: 23-40 years) and 9 CHD patients (mean age: 5.9 years, range: 0.6-21 years), 3D flow data were then acquired using a nominal acceleration factor of 8 with 11 and 7 training profiles along both phase encoding directions and reconstructed using *k-t* PCA. Partial Fourier sampling was not used. The v_enc_ was chosen to match the expected peak velocity in the heart and surrounding vessels. The entire heart and all surrounding vessels of interest were covered by the field-of-view. In all subjects, prospective ECG triggering was used. Scan parameters are listed in Table 1*.* In volunteers, 24 heart phases corresponded to an acquired temporal resolution of 35.6 ± 5.3 ms (no temporal interpolation was used). Breathing motion was monitored using a pencil-beam navigator placed on the dome of the right hemi-diaphragm played out at the beginning of each ECG cycle. A gating window of 3-5 mm was used resulting in navigator efficiencies on the order of 40-50% in volunteers and, due to a more regular breathing during general anaesthesia, of 50-70% in patients.

In all volunteers and patients, time-resolved 2D through-plane encoded flow data were acquired during free breathing and with retrospective ECG gating using a clinically validated acquisition protocol [25]. To reduce breathing motion artefacts, 2-3 signal averages were acquired in all time-resolved 2D flow acquisitions. Sequence parameters further included: TR/TE = 4.5/2.8 ms, spatial resolution = 2.5×2.5 mm^2^, 30 heart phases, slice thickness: 7 mm, Flip angle: 15 deg. The v_enc_ was chosen to match the maximum velocity in the vessel of interest. In volunteers, flow was quantitatively assessed in the ascending aorta (AAo), the main branch pulmonary artery (MPA), the left and right pulmonary arteries (LPA and RPA) and the superior vena cava (SVC). In patients, the area of flow quantification varied depending on the CHD type.

### Reconstruction

Clinically validated time-resolved 2D flow data were reconstructed on-line and included concomitant field and eddy current correction provided by the manufacturer.

Time-resolved 3D flow data were reconstructed using *k-t* PCA [23] in combination with a sparsifying transform [17]. To this end, the fully sampled low-resolution training and the undersampled data were transformed using a complex difference operator prior to *k-t* PCA reconstruction. Subsequently, data were transformed into their spatial-temporal frequency representations and temporal basis functions were derived from the training data using principal component analysis (PCA). Data unfolding was performed using a weighted least-squares approach [23]. Resulting phase maps were corrected for concomitant field and eddy current related phase offsets [26-28]. The reconstruction code was implemented in C and reconstruction times for 3D flow data were on the order of 2 minutes on a 12 core CPU cluster, depending on matrix size, number of heart phases and number of coil elements.

For comparison, the retrospectively undersampled reference dataset was also reconstructed using *k-t* SENSE.

### Post-processing

Images were analysed offline using GTFlow (GyroTools LLC, Zurich, Switzerland). Contours were manually drawn to segment vessels of interest. In order to avoid misalignment due to subject motion all vessels were contoured separately on the 3D flow datasets. Flow curves and stroke volumes derived from time-resolved 3D and 2D flow data were then compared.

### Data analysis

#### Retrospective undersampling

The time-average of the cumulated absolute flow rate error *E*_*R*_ (in ml/s) with respect to the reference dataset was calculated for different reconstruction factors R:

$$E_{R}=\frac{1}{n_{p}}\sum_{t=0}^{t_{n_{p}}}\left|Q_{1}\left(t\right)-Q_{R}\left(t\right)\right|$$

Where *Q*_1_(t) and *Q*_*R*_(t) correspond to the flow rates through the vessel of interest in the dataset with a *k-t* undersampling factor of 1 and *R* respectively at the time-point t. *n*_*p*_ corresponds to the number of time-frames. This error metric provides the cumulated flow error (over time), making it more sensitive to potential undersampling artefacts such as temporal blurring.

Particle trace analysis was performed by counting the percentage of particles emitted from a contour within the ascending aorta and reaching a second contour in the descending aorta.

#### Prospective undersampling

Flow rates from undersampled time-resolved 3D flow datasets were compared to time-resolved 2D flow rates by quantifying stroke volumes in the vessels of interest. The error in [ml] was calculated as:

$$E_{\mathrm{SV}}=\sum_{t=0}^{n_{p,2D}\Delta t_{2D}}\Delta t_{2D}Q_{2D}\left(t\right)-\sum_{t=0}^{n_{p,3D}\Delta t_{3D}}\Delta t_{3D}Q_{3D}\left(t\right)$$

Where *Q*_*2D*_(t) and *Q*_*3D*_(t) correspond to flow rates of the time-resolved 2D and 3D flow data and ∆*t*_*2D*_, ∆*t*_*3D*_ to their temporal resolutions.

As the peak flow rate is expected to be more sensitive to temporal blurring, the values extracted from the volunteers’ datasets in the ascending aorta were also compared between 2D and 3D acquisitions.

In CHD patients, the time-resolved 3D flow datasets were further qualitatively analysed using particle path visualisation.

## Results

### Retrospective undersampling

**Figure 1:**
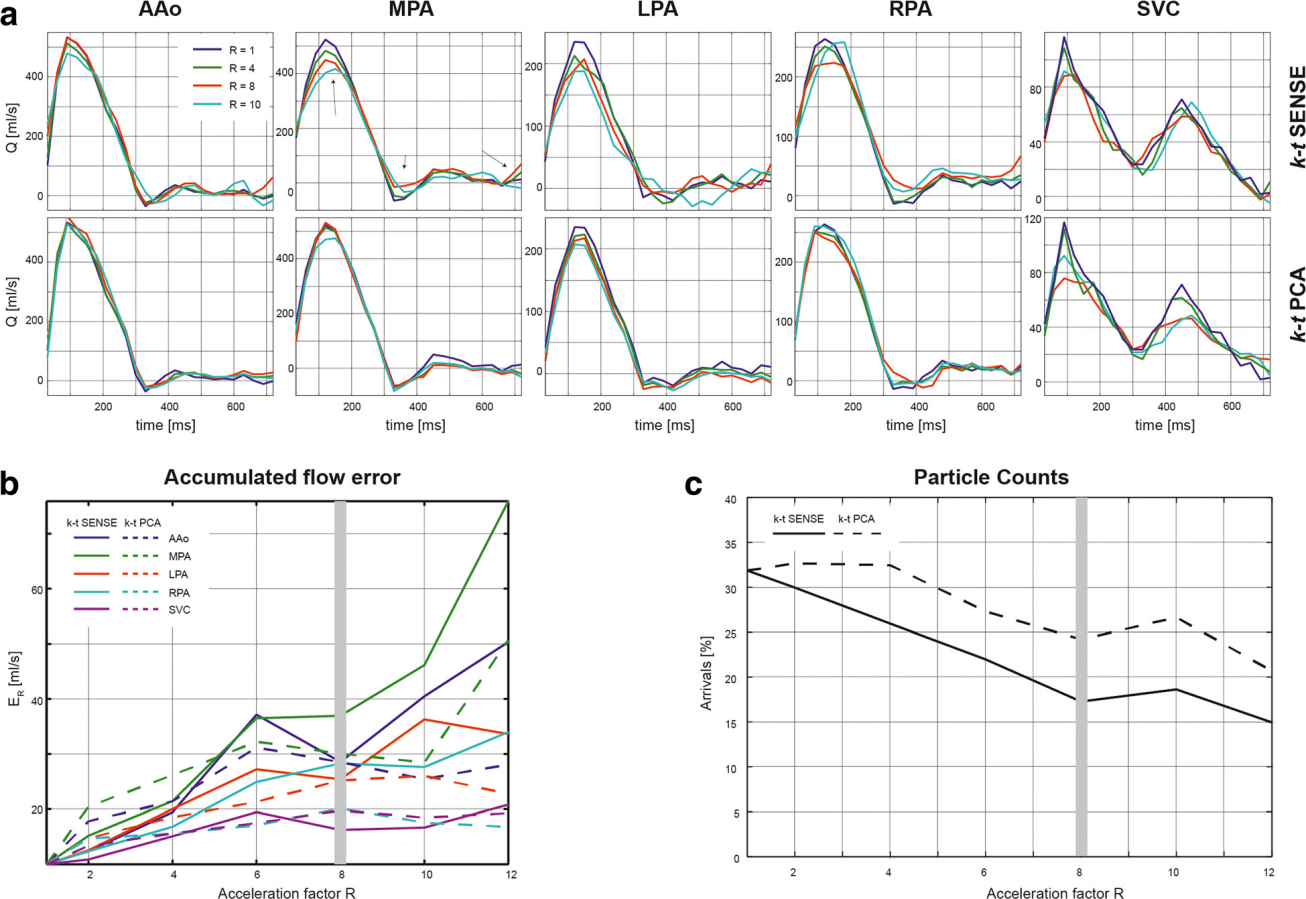
**Undersampling simulation results. a)** Retrospectively undersampled and reconstructed data using *k-t* SENSE and *k-t* PCA for different acceleration factors along with data from the fully sampled reference (R = 1). **b)** Accumulated flow errors as a function of the acceleration factor for *k-t* SENSE (full lines) and *k-t* PCA (dotted lines). An acceleration factor of 8 (grey vertical line) was chosen for all prospective acquisitions. **c)** Percentage of particles ejected from the ascending aorta reaching the descending aorta as a function of the acceleration factor.

Figure 1a shows flow curves retrospectively undersampled with a *k-t* acceleration factor of R = 4, 8 and 10 along with the reference flow curve (R = 1). Datasets were reconstructed using *k-t* SENSE (top row) and *k-t* PCA (bottom row). The accumulated flow error for both reconstructions as a function of the acceleration factor is shown in Figure 1b. Figure 1c shows the percentage of particles detected at the level of the descending aorta, emitted from the ascending aorta as a function of the undersampling factor for *k-t* SENSE and *k-t* PCA reconstructions.

### Volunteer study

**Figure 2:**
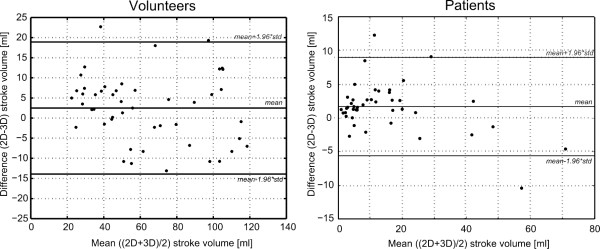
**Bland-Altman stroke volume analysis.** Bland-Altman plot comparing stroke volumes extracted from 2D flow and highly accelerated 3D flow data in volunteers (left) and patients (right).

A correlation and Bland-Altman analysis of all stroke volumes derived from 2D flow acquisitions and undersampled 3D flow data reconstructed using *k-t* PCA are shown in Figure 2. Linear regression resulted in a correlation coefficient of R^2^ = 0.93 and the Bland-Altman analysis revealed an underestimation of stroke volume by 2.5 ± 8.4 ml with 3D flow corresponding to 5.6 ± 14.9% with respect to the stroke volumes derived from the 2D flow datasets. Peak flow rates from the 3D datasets showed an underestimation by 5.1 ± 7.5% with respect to the 2D datasets.

### CHD patients

Flow comparison between stroke volumes extracted from time-resolved 2D flow and undersampled time-resolved 3D flow reconstructed using *k-t* PCA revealed an underestimation using 3D flow of 1.6 ± 4.8 ml summarising all vessels which corresponded to 18.1 ± 33.3% of the stroke volumes derived from the time-resolved 2D flow data (Figure 2). Vessels with a stroke volume over 20 ml showed a deviation of 2.8 ± 14.5% and vessels with a stroke volume under 20 ml showed a deviation of 23.4 ± 36.4%.

### Qualitative analysis

**Figure 3:**
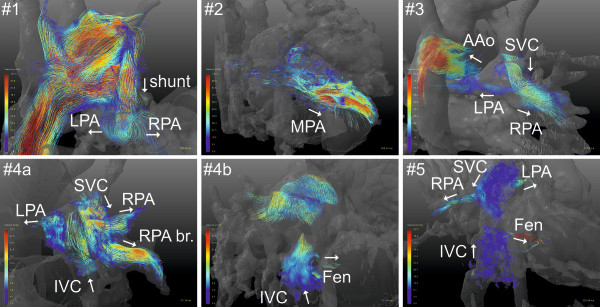
**Pathline screenshots.** Particle traces ejected from different vessels of interest in 5 CHD patients show different flow patterns (see text for detail). Corresponding movies can be found in the Additional files.

As shown in Table 1, the present study contained 5 different types of CHDs: 7 patients with Hypoplastic Left Heart Syndrome (HLHS), one patient with Tetralogy of Fallot (ToF) and one patient with a Double Inlet Left Ventricle (DILV). In patients with HLHS and DILV, similar surgery was performed consisting of the Norwood I procedure (Blalock-Taussig shunt connecting the left subclavian artery with the pulmonary arteries), followed by a procedure leading to the Hemifontan stage (grafting of the SVC onto the pulmonary arteries) and the final surgery leading to the Fontan stage (connection of SVC and IVC with the pulmonary arteries). These stages are denoted by I-III in Table 1. Figure 3 shows screenshots of particle trace visualisations in patients of each CHD category and stage of Fontan procedure. Corresponding movies are included in the Additional files 1, 2, 3, 4, 5, and 6. Patient numbers correspond to the numbering in Table 1. In each figure, the main vessels of interest as well as main blood flow directions (arrows) are annotated. Patient #1 with Hypoplastic Left Heart Syndrome (HLHS) in the first stage of surgery shows strong circular flow from the Blalock-Taussig shunt into the RPA and LPA. It shows that the main fraction of emitted particles is ejected into the RPA leading to an uneven distribution between RPA and LPA flow volumes. Particles released from the AAo are further observed to enter the subclavian artery and the shunt with velocities reaching 2 m/s. Patient #2 (Tetralogy of Fallot) shows severe pulmonary regurgitation during early diastole. In patient #3 (HLHS, Hemifontan) circular flow is observed at the level of the branching of the pulmonary arteries. The bulk flow from the SVC follows a laminar flow into the RPA leading to an uneven flow distribution between LPA and RPA. Particles ejected from the AAo further show a circular flow pattern due to the connection of native- and neo-aorta. Patient #4 (HLHS, fenestrated cavo-pulmonary connection Fontan) shows an uneven flow distribution into RPA and LPA (bulk flow into the RPA) and circular flow into the *Fontan* branching of SVC, IVC, RPA and LPA. Particles ejected below the level of the fenestration show systolic flow through the fenestration into the left atrium. In patient #5 (Double Inlet Left Ventricle (DILV) with fenestrated cavo-pulmonary connection *Fontan*) high velocity (1 m/s) flow is observed through the fenestration. Similar to patient #4, circular flow is observed into the *Fontan* circulation due to the confluence of flow from SVC and IVC being redirected into LPA and RPA.

## Discussion

The presented results demonstrate the potential of highly accelerated time-resolved 3D flow in a clinical setting. In patients, the nominal scan times of the time-resolved 3D flow acquisition covering the entire heart and great vessels were on the order of 6 min resulting in a total net scan time of 8 min depending on breathing navigator efficiency and cardiac frequency. Image reconstruction times were below 3 min and hence the overall protocol could be well established during clinical workflow. Although similar scan times have been achieved recently, previous data were acquired with a smaller field of view [15], lower temporal resolutions in combination with interpolation [18] or with a 100% gating efficiency tolerating breathing motion artefacts [29].

The results also show that *k-t* PCA is able to reconstruct highly undersampled data even with low receive channel count (5-channel coil used in volunteers, 2-5 channel coils used in patients) providing flexibility in selecting appropriate coils also in smaller and young patients. While the channel count is not critical in the present application, it is noted that large coil arrays are becoming increasingly available for frame-to-frame parallel imaging methods.

A nominal acceleration factor of 8 was used although the retrospective results in this study showed that using *k-t* PCA with an undersampling factor of 10 might also be feasible. In volunteers, flow curves compared well between accelerated time-resolved 3D flow data and time-resolved 2D flow data with stroke volume deviations of 2.5 ± 8.4 ml in line with previous studies [2,30].

*k-t* BLAST was not assessed in this study as it is known to be more susceptible to temporal blurring [10,11].

Besides the quantitative validation, the present study has also demonstrated that valuable qualitative hemodynamic patterns can be extracted from the time-resolved 3D flow dataset.

Flow rates are known to be very sensitive to global phase offsets [31]. Although care has been taken to correct for eddy current related phase offsets, a limited signal to noise ratio and lack of static tissue in some areas can compromise background phase fitting and interpolation. Phase offsets affect both time-resolved 2D flow and 3D flow in different ways due to differences in sequence parameters [32].

Another potential source of error is attributed to the delineation of vessel contours [33,34]. As magnitude image contrast is often reduced in time-resolved 3D flow data mainly due to the lack of inflowing unsaturated blood, a correct delineation of the vessel border can be challenging, especially for small, venous vessels.

An important drawback of the four-point time-resolved 3D flow acquisitions lies in the limited velocity-to-noise ratio in vessels with small velocities [35]. Since the value of the v_enc_ has to be set to match the maximum expected velocity in the entire volume, smaller velocities are measured with less accuracy. This is reflected in the large standard deviation of over 30% of the stroke volumes as measured in the patients if all vessels were included in the analysis. If only vessels with a stroke volume larger than 20 ml are considered, the standard deviation is reduced to 15.2%. Errors in the high flow vessels (mainly arterial) therefore agree well with findings in volunteers. Especially in CHD patients, the velocity range in the different vessels can be very large. By using a multi-point acquisition [36,37] at the expense of longer scan times, this drawback can be alleviated leading to higher velocity-to-noise ratios in vessels with low flow.

Finally, since prospective triggering and navigator based gating was used for 3D flow CMR, the first and last 20 ms of early systole and late diastole could not be sampled. The impact on stroke volume calculations is especially important when high flow is present in early systole or late diastole and in cases with strong cardiac cycle variability. The use of self-navigation techniques in combination with a retrospective k-t sampling scheme might alleviate this issue.

## Conclusions

In summary, this work has demonstrated that the acquisition and reconstruction of undersampled Cartesian 3D flow CMR is feasible in a clinical setting. The straight-forward planning process of time-resolved 3D flow acquisition is of great value in complex CHD anatomies.

## Abbreviations

PCA: Principal component analysis; BLAST: Broad-use linear acquisition speed-up technique; SENSE: SENSitivity encoding; CHD: Congenital heart disease; HLHS: Hypoplastic left heart syndrome; DILV: Double inlet left ventricle; AAo: Ascending aorta; DAo: Descending aorta; MPA: Main pulmonary artery; RPA: Right pulmonary artery; LPA: Left pulmonary artery; SVC: Superior vena cava; IVC: Inferior vena cava; Fen: Fenestration (shunt from Fontan tunnel into right atrium).

## Competing interests

The authors declare that they have no competing interests.

## Authors’ contributions

DG designed the study, co-developed the acquisition and reconstruction algorithms, collected and analysed the data and drafted the manuscript. JW contributed to the study design, to the data acquisition and analysis and to patient recruitment. GG recruited the patients and contributed to the study design. MB co-implemented the data reconstruction framework. TS contributed to the design of the study and supervised the work. SK contributed to the design of the manuscript and supervised the work. All authors read and approved the final manuscript.

## Supplementary Material

Additional file 1 **Pathline movie - Norwood I procedure.** Posterior view of the aortic arch including subclavian artery and Blalock-Taussig-Shunt of patient 1 (HLHS after Norwood I procedure). Particles are released in the ascending aorta and the shunt. Click here for file

Additional file 2 **Pathline movie – Tetralogy of Fallot.** Antero-lateral view of the main pulmonary artery of patient 2 (Tetralogy of Fallot). Particles are released in the main pulmonary artery during the entire cardiac cycle. Click here for file

Additional file 3 **Pathline movie - Hemifontan.** Posterior view of the thoracic aorta, superior vena cava and left and right pulmonary arteries of patient 3 (HLHS Hemifontan). Particles are release in the ascending aorta and all three branches of the Hemifontan circulation. Click here for file

Additional file 4 **Pathline movie – Fontan (a).** Posterior view of the Fontan circulation in patient 4 (HLHS with fenestrated cavo-pulmonary connection Fontan). Particles are released in the superior vena cava, inferior vena cava and the left and right pulmonary arteries. Click here for file

Additional file 5 **Pathline movie – Fontan (b).** Anterior view of the Fontan circulation in patient 4 (HLHS with fenestrated cavo-pulmonary connection Fontan). Particles are released below the fenestration in the inferior vena cava as well as inside the Fontan connection. Click here for file

Additional file 6 **Pathline movie – DILV Fontan (1a).** Anterior view of the Fontan circulation in patient 5 (DILV with fenestrated cavo-pulmonary connection Fontan). Particles are released below the fenestration in the inferior vena cava as well as in the superior vena cava. Click here for file
